# Endometrial Organoids and Their Role in Modeling Human Infertility

**DOI:** 10.3390/cells14110829

**Published:** 2025-06-03

**Authors:** Abdullah Jabri, Mohamed Alsharif, Tasnim Abbad, Bader Taftafa, Abdulaziz Mhannayeh, Abdulrahman Elsalti, Fayrouz Attia, Tanveer Ahmad Mir, Islam Saadeldin, Ahmed Yaqinuddin

**Affiliations:** 1 College of Medicine, Alfaisal University, Riyadh 11533, Saudi Arabia; ajabri@alfaisal.edu (A.J.); moalsharif@alfaisal.edu (M.A.); tabbad@alfaisal.edu (T.A.); betaftafa@alfaisal.edu (B.T.); amohanaya@alfaisal.edu (A.M.); fattia@alfaisal.edu (F.A.); tmir@kfshrc.edu.sa (T.A.M.); imohamed@kfshrc.edu.sa (I.S.); 2 International School of Medicine, Istanbul Medipol University, Istanbul 34810, Turkey; abdulrahman.el@std.medipol.edu.tr; 3 Laboratory of Tissue/Organ Bioengineering & BioMEMS, Organ Transplant Centre of Excellence (TR&I-Dpt), King Faisal Specialist Hospital & Research Centre, Riyadh 11211, Saudi Arabia; 4 Comparative Medicine Department, King Faisal Specialist Hospital & Research Centre, Riyadh 11211, Saudi Arabia

**Keywords:** endometrial organoids, 3D culture, endometrial receptivity, implantation failure, infertility

## Abstract

Endometrial organoids (EOs) have emerged as a powerful three-dimensional (3D) model for studying the human endometrium, offering new insights into infertility and reproductive disorders. These self-organizing miniature structures closely mimic the cellular composition, hormonal responsiveness, and functional characteristics of the endometrium, making them valuable preclinical tools for investigating implantation failure, endometrial receptivity, and disease pathophysiology. This review explores the role of EOs in reproductive medicine, with a focus on their applications in infertility research, environmental toxicology, and regenerative therapies. Traditional 2D cell cultures fail to capture the complexity of these physiological and pathological interactions, whereas organoids provide a physiologically relevant system for studying implantation mechanisms. Additionally, co-culture models incorporating stromal and immune cells have further enhanced our understanding of the maternal–fetal interface. Beyond modeling infertility, EOs hold significant promise for therapeutic applications. Advances in organoid transplantation have demonstrated potential for treating endometrial dysfunction-related infertility, including conditions such as Asherman’s syndrome and thin endometrium. Moreover, these models serve as a platform for drug screening and biomarker discovery, paving the way for personalized reproductive medicine. Despite their transformative potential, limitations remain, including the need for improved extracellular matrices, vascularization, and immune system integration. This review emphasizes the significant contributions of EOs to the field of infertility treatment and reproductive biology by examining recent advancements and emerging research. The continued refinement of these models would offer a paradigm for improving assisted reproductive technologies (ARTs) and regenerative medicine outcomes, offering new hope for individuals facing infertility challenges.

## 1. Introduction

One of the major advances that has revolutionized the field of biomedical research is the invention of organoids. Organoids are three-dimensional (3D) in vitro structures formed from cell clusters that are capable of self-organization and differentiation into different cell types [1]. They are considered to be mini-organs as they possess cellular heterogeneity and exhibit functional characteristics of organs they resemble in vivo. The term “organoid” gained popularity in the field in 2009 when Clevers et al. were able to generate intestinal organoids capable of self-organization and differentiation into crypt-villus structures from single leucine-rich repeat containing G protein-coupled receptor 5 (Lgr5)-expressing adult intestinal stem cells [2]. Nevertheless, the history of organoids traces back to the 1900s, when Wilson described his attempt of in vitro regeneration by showing that dissociated sponge cells can self-organize to generate a whole organism [3]. These miniature organ models have been used to study disease mechanisms, drug screening, and regenerative medicine. Organoids mimic the normal behavior of tissues and organs, providing a baseline for understanding organ-specific biology. However, their ability to replicate pathological features makes them indispensable for studying diseases. For example, organoids can model disease mechanisms by mimicking the way normal cellular processes are disrupted in response to genetic mutations, environmental stressors, or other pathological stimuli. By comparing healthy organoids to diseased ones, researchers can uncover the underlying mechanisms of diseases such as cancer, liver cirrhosis, and neurological disorders. Various organoids have been developed, including those representing the intestine, liver, brain, and reproductive system, each providing insights into organ-specific biology and pathology [4,5,6]. Among these, endometrial organoids (EOs) have emerged as invaluable models for studying the human endometrium. The endometrium is a highly dynamic tissue that goes through periodic changes in which it becomes receptive to embryo implantation—a process crucial for fertility and successful pregnancy [7]. Though the dysregulation of endometrial receptivity is a major contributor to infertility, a lot remains to be answered. Therefore, Eos, through their unique characteristics, serve as a novel platform for investigating implantation failures and other reproductive disorders. One such application involves investigating the impact of environmental toxins—such as Bisphenol A (BPA)—which have been shown to impair endometrial function and contribute to infertility. Recent studies using EOs as a toxicology model have revealed that BPA exposure induces oxidative stress, apoptosis, and metabolic dysfunction in endometrial tissue, ultimately compromising fertility [8]. Traditional 2D cell cultures fail to capture the complexity of these disruptions, making 3D organoids a valuable tool for studying environmental toxicants and their effects on reproductive health.

In this review, we aimed to explore the role of EOs in understanding and addressing infertility by examining how these organoids contribute to the study of endometrial receptivity and implantation and shed light on the molecular and cellular factors that influence successful embryo attachment. Moreover, we highlight the potential of organoid transplantation as a therapeutic strategy for endometrial dysfunction-related infertility. By integrating current knowledge and future directions, this review aims to focus on the transformative impact of EOs in reproductive medicine and their potential to advance infertility treatments.

## 2. Endometrial Receptivity and Implantation

**Figure 1:**
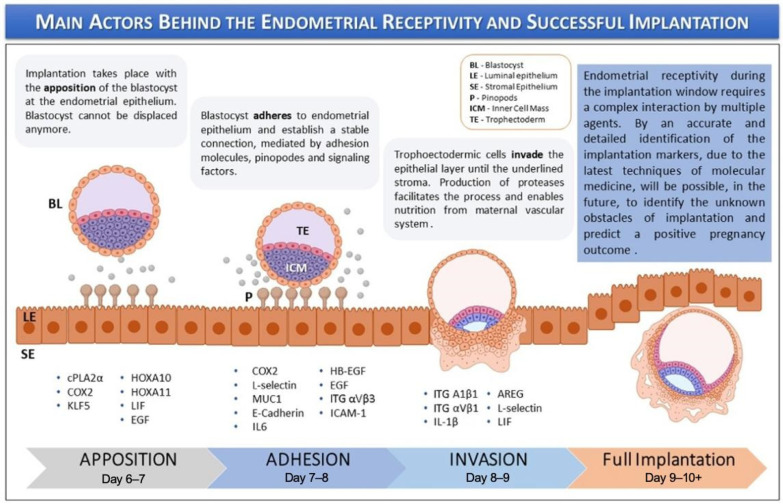
Keys actors behind the endometrial receptivity and implantation. The image is adapted from Governini et al., 2021, with copyright permission [10].

Endometrial receptivity is defined as the period when the endometrium permits the trophectoderm of the blastocyst to attach and invade (Figure 1). This receptive phase is tightly regulated by estrogen and progesterone; the latter plays a crucial role in promoting anti-inflammatory actions, immune tolerance, and receptor downregulation, all of which ensure timely implantation and the maintenance of early pregnancy. Receptivity also includes key molecular events like the downregulation of estrogen receptor-alpha (ER-α) and the expression of progesterone receptors (PRs). In women with regular menstrual cycles, the window of receptivity occurs during the mid-luteal phase of the menstrual cycle, approximately 6 to 10 days after the luteinizing hormone (LH) surge, although the precise timing may vary depending on cycle length and hormonal dynamics [9].

The implantation process is divided into three sequential phases: (i) apposition, (ii) adhesion, and (iii) invasion. During these stages, numerous molecular mediators—regulated by ovarian steroid hormones—facilitate the initial maternal–fetal interaction. These include adhesion molecules, cytokines, growth factors, lipids, and other signaling factors [10]. Key markers of endometrial receptivity include endometrial thickness, volume, and morphological appearance. EOs effectively model the cellular environment necessary for implantation by mimicking the structural and functional features of the endometrial epithelium, which undergoes essential transitions during receptivity. Derived from human endometrial epithelial cells, these organoids self-organize into three-dimensional, gland-like structures and exhibit apicobasal polarity similar to that of native endometrial glands. This polarity allows for the separate collection of apical and basal secretions to investigate their respective roles in implantation [6].

EOs closely resemble the mid-secretory phase of the menstrual cycle and exhibit strong responsiveness to hormones like progesterone and estrogen [11]. This includes the upregulation of key receptivity markers such as Heat Shock Protein A9 (HSPA9), which enhances epithelial adhesiveness for blastocyst attachment, and Dipeptidyl Peptidase 4 (DPP4), which promotes glandular development [12]. Furthermore, organoids release apical secretions into their inner lumen that simulate uterine fluid, containing proteins like DPP4 and HSPA9. Dysregulation of these indicators has been observed in organoids derived from infertile patients through high-throughput screening of these secretions, providing a model to investigate causes of faulty receptivity [11].

The basal secretions of EOs resemble the communication between stromal and epithelial cells, by replicating stromal signals necessary for implantation [13]. For instance, EOs secrete extracellular vesicles carrying proteins like cystatin C, which controls stromal decidualization, and microRNAs like miR-92a-3p, which modulate receptivity. Organoids can also be modified to form epithelial monolayers that mimic the luminal epithelium—a surface required for blastocyst attachment. In conjunction with Wnt inhibitors, hormonal therapies improve blastocyst adhesion and allow for the functional modeling of contraceptive drugs by increasing the expression of receptivity genes such as Leukemia Inhibitory Factor (LIF), DPP4, and glutathione peroxidase 3 [14].

## 3. Development, Structure, and Formation Methods of Organoids

Endometrial epithelial cells are isolated and cultured to create EOs; the development and upkeep of these structures rely on particular growth factors and extracellular matrices [15,16,17,18,19]. Unlike conventional 2D cultures, which lack cellular diversity and long-term viability, 3D EOs provide a more physiologically relevant system for studying uterine function. Recent research highlights how organoids derived from fertile and infertile patients exhibit distinct hormonal responses, making them useful for implantation and infertility studies. Additionally, microfluidic systems have been introduced to enhance the study of maternal–embryonic interactions, offering a new frontier for implantation failure research [20].

**Figure 2:**
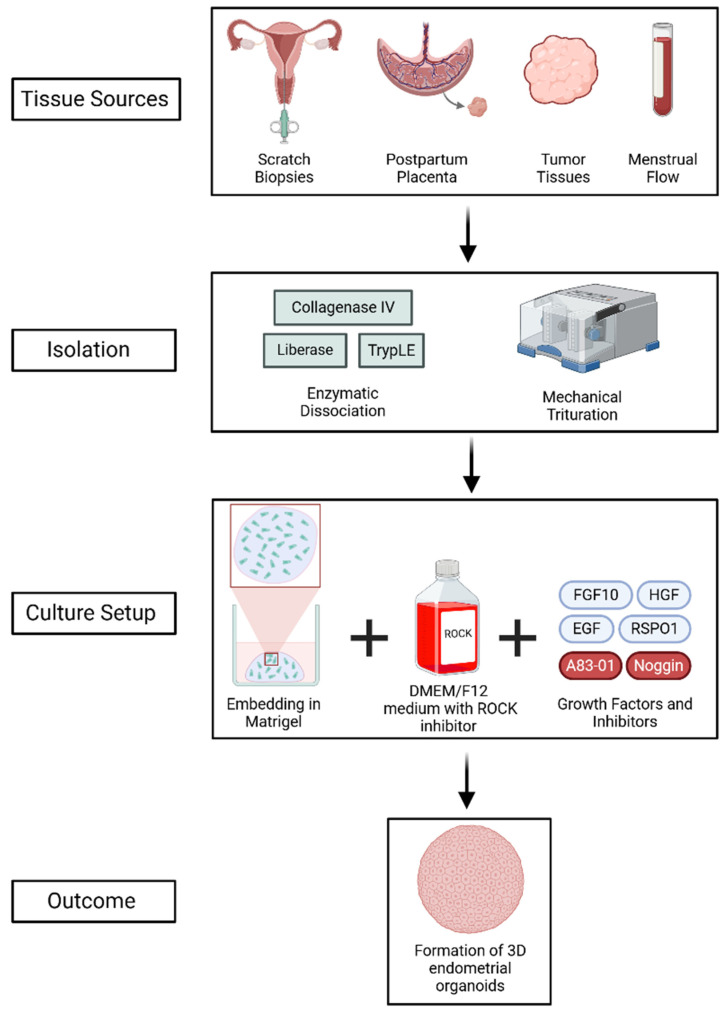
Generation of 3D EOs from various tissue sources through enzymatic and mechanical dissociation, followed by culture in Matrigel with growth factors and inhibitors. The process supports self-renewal and mimics the endometrial epithelium. Created using BioRender (web-based tool, https://www.biorender.com/). Yaqinuddin, A. (2025). Retrieved from https://BioRender.com/4fap8ty (accessed on 13 February 2025).

EOs can be derived from a variety of sources, such as postpartum placenta, tumor tissues, scratch biopsies, and menstrual flow (Figure 2) [17,18,19]. Typically, patient-derived samples are enzymatically dissociated using agents such as collagenase IV, TrypLE, or liberase, followed by mechanical trituration to digest the tissues and generate endometrial and endometriotic organoids [15,16,21]. The isolated epithelial cells are then embedded in a 3D matrix, such as Matrigel, and cultured in advanced DMEM/F12 medium supplemented with a ROCK inhibitor to promote cell proliferation and survival. The culture media is additionally enriched with vital growth factors, such as fibroblast growth factor 10 (FGF10), hepatocyte growth factor (HGF), epidermal growth factor (EGF), and R-spondin-1 (RSPO1), which activate important signaling pathways like MAPK and Wnt/β-catenin [22,23,24,25]. Additional components such as Noggin and the TGF-β inhibitor A83-01 promote self-renewal by inhibiting BMP and TGF-β signaling, respectively [26,27,28,29,30,31].

Similar to native endometrial glands, EOs are spheroidal structures surrounded by a single columnar epithelium [32]. A mix of epithelial markers, such as EPCAM, PanCK, and E-cadherin, preserves their structural integrity. Progenitor cells (LRIG1, PROM1, AXIN2, SOX9), epithelial cells (EPCAM, KRT7, CDH1, SOX17), secretory cells (PAEP, MUC1, PAX8, HSD17B2), and ciliated cells (FOXJ1, PIFO, RSPH1) are among the several cellular subtypes of EOs that have been identified through genetic studies [11,19,33,34,35].

**Figure 3:**
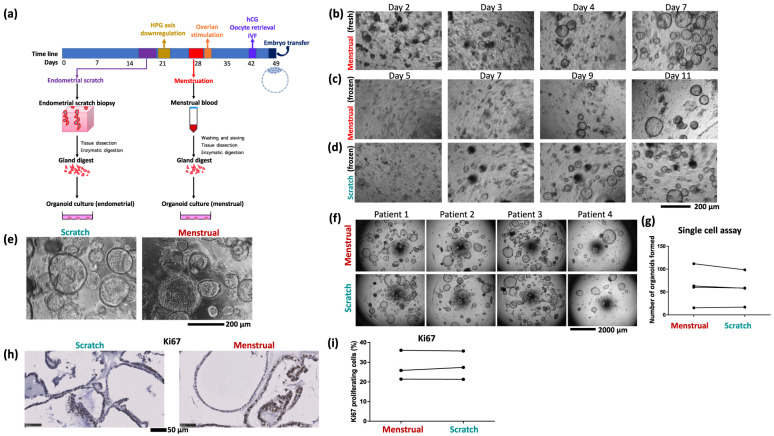
(**a**) Schematic of the organoid culture process using samples of endometrial scratch and menstrual blood from the same cycle. (**b**) Growth of menstrual organoids obtained directly from menstrual blood. (**c**) Growth of organoids from frozen menstrual blood, (**d**) and scratch, samples of the same patient. (**e**) Organoids derived from endometrial scratch and menstrual blood of the same patient are morphologically indistinguishable. Image shows organoids seeded from a single cell (5000 cells per 20 µL Matrigel drop) after 9 days of growth. (**f**) Single cell assay comparing growth of paired menstrual organoids and scratch organoids derived from four patients. (**g**) Quantification of single cell assay. Quantified 5 to 8 well repeats per condition. (**h**) Representative images of immunostaining for the proliferation marker Ki67 in menstrual organoids and scratch organoids from one patient. (**i**) Quantification of Ki67 proliferation index in menstrual organoids and scratch organoids of three patients. The image is adopted from Cindrova-Davies Tazangi et al., 2021, with copyright permission under the terms of the CC BY NC ND 4.0 license [18].

Depending on the tissue source and the goals of the investigation, various techniques are used to produce EOs (Figure 3). For example, endometrial samples from hormonally untreated patients undergoing laparoscopy for benign gynecological disorders are mechanically triturated, enzymatically dissociated with collagenase IV, and cultured in specialized media within a 70% Matrigel matrix [21]. After being digested by collagenase I, DNase, and dispase, cell suspensions obtained from premenopausal hysterectomy samples are filtered and cultivated in MammoCultTM medium under epithelial–stromal co-culture conditions [36]. Likewise, single-cell suspensions of human endometrial and decidual samples can be prepared, embedded in hydrogels such as TeloCol-6, and maintained in an ideal organoid culture medium with regular media changes every two days [16,37].

Organoid development is monitored over a 20–30-day period after single epithelial cells are implanted in Matrigel to determine clonogenic potential [15]. Periodic passaging every 10–20 days ensures the expansion and viability of EOs for biobanking purposes and allows for long-term maintenance [21]. Recent advances in synthetic hydrogel formulations that mimic the endometrial extracellular matrix have enhanced physiological relevance by improving organoid stability and epithelial–stromal interactions [38,39,40,41,42,43,44,45,46].

## 4. Organoid Transplantation as a Therapeutic Strategy for Infertility

Endometrial organoid transplantation (EOT) is one of the regeneration techniques being investigated because conventional treatments like hormone therapy or surgical adhesiolysis often fail to restore functioning endometrial tissue [47]. EOT gives patients with refractory infertility hope by promoting structural regeneration, improving vascularization, and improving reproductive outcomes [48].

EOT has demonstrated exceptional effectiveness in restoring endometrial thickness and lowering fibrosis in animal models of endometrial damage. In rats, for instance, endometrial thickness was recovered to 476.80 ± 54.26 µm after receiving a transplant of Müllerian duct-like cells (MDLCs) produced from human pluripotent stem cells (hPSCs), greatly exceeding that of untreated controls [49]. Similarly, He et al. highlighted the role of angiogenesis in tissue repair by observing decreased fibrotic areas and increased vascular endothelial growth factor A (VEGFA) expression after transplanting stage-specific embryonic antigen-1 positive (SSEA-1+) endometrial epithelial stem cells [50]. Increased micro-vessel density and VEGF expression were noted in treated mice, indicating enhanced vascularization, which is essential for embryo implantation [49]. Moreover, transferring mitochondria from organoids to host cells is a unique repair strategy that improves cellular metabolism and repair. These structural improvements correlate with functional outcomes, including restored hormonal responsiveness and decidualization capacity—key processes for embryo implantation [49].

The therapeutic potential of EOT is further validated by pregnancy outcomes. Zhang H et al. reported successful pregnancies in three out of four mice with intrauterine adhesions (IUAs) post-transplantation [51]. Similarly, Gong et al. reported an 83.33% pregnancy rate in rats treated with MDLC, compared to 50% in injured controls [49]. Litter size improvements were also observed; for example, mice receiving endometrial mesenchymal stem cell spheroids had an increase from roughly three to five pups [52].

Clinically, EOT shows potential for certain causes of infertility. According to Wiweko, autologous cell-based therapies for thin endometrium can improve endometrial receptivity by promoting epithelial regeneration and E-cadherin expression [53]. Another use is postoperative recovery; Park et al. used 3D stem cell-laden constructions to achieve live births in endometrial ablation models, with no morphological or chromosomal abnormalities seen in the offspring [54].

## 5. Endometrial Organoids as Models for Infertility and Endometrial Diseases

EOs have revolutionized endometrial research by providing a 3D model that mimics the in vivo endometrium. Derived from human endometrial tissue, these organoids can mimic key structural and functional features of the endometrium, making them significantly useful for studying diseases like endometriosis, endometrial cancer, and infertility. Infertility is clinically defined as the failure to achieve pregnancy after 12 months or more of regular, unprotected intercourse [55]. Around one in six couples are affected by infertility globally, with about 30% of the cases attributed solely to the female factor [56]. Traditionally, animal in vivo models have been used to model the human endometrium [57]. Although these studies provided important insights, they did not accurately model the human endometrium [58]. Hence, functional in vitro studies of the human endometrium are needed to explore the different causes and conditions of infertility.

### 5.1. Endometriosis

Endometriosis is a chronic estrogen-dependent condition where functional tissue lining the uterus grows outside the uterine cavity [59]. The main symptoms are chronic pelvic pain and infertility. Endometriosis is a big health issue that is estimated to affect between 5 and 15% of women of reproductive age in the United States and Canada [60]. It is considered incurable, and, despite extensive research, no cause has been found; only theories exist. This situation highlights the need for new investigative and therapeutic approaches, like EOs. The first endometriosis organoid model was cultured in 2013, which more accurately mimicked the molecular and histological features of endometriosis compared to the previous 2D models [61]. Further models also confirmed the similarity between EOs and endometrial tissue [15,62]. Boretto et al. developed an organoid of eutopic and ectopic endometriosis that possessed the typical features of endometriosis and cancer-linked mutations [15]. Eshfendiari et al. observed significant alterations in methylation levels of *HOX* clusters and cofactors in both ectopic and eutopic endometrial tissues, as well as in the corresponding organoids, compared to normal endometrial tissue [62]. These findings concluded that EOs can maintain the epigenetic changes and thus can be used to study endometriosis further. Later, the research group established another endometriosis organoid model that showed that progesterone receptor B (*PR-B*) is downregulated in ectopic and eutopic EOs compared to healthy controls, yet through different mechanisms [63]. Although eutopic endometriosis organoids exhibited the hypermethylation of *PR-B* and ectopic endometriosis organoids showed the hypomethylation of *PR-B*, it was downregulated anyhow. This inconsistency suggests that other epigenetic mechanisms, such as histone modifications or micro-RNAs (miRNAs), may be responsible for the downregulation of *PR-B* in endometriosis.

### 5.2. Endometrial Dysfunction

In 2020, Bui et al. established organoid lines from cryopreserved endometrial tissue obtained from infertile women [64]. Following this, several studies used organoids to compare the endometrium of fertile and infertile women [65,66,67]. They noted the upregulation of cell cycle processes during the secretory phase of the infertile organoids. Moreover, it was found that there was increased proliferative activity in the glandular cells of the endometrium in infertile women compared to fertile women. This high proliferative activity in the secretory phase can lead to inadequate differentiation and a poorly receptive endometrium for embryo implantation. Conversely, another study has shown the downregulation of the cell cycle in the luteal phase in the infertile endometrium [68]. These findings suggest the cell cycle is altered in different ways in the infertile endometrium, which indicates that no one cause of infertility applies to all. One of the key applications of EOs is their ability to model implantation disorders. Studies have shown that fertile vs. infertile EOs differ in their response to estrogen and progesterone, suggesting that implantation failure may stem from underlying endometrial receptivity defects [20]. Furthermore, microfluidic-based organoid models now allow for the study of trophoblast attachment and early embryo interactions, making them valuable for research into maternal–fetal health [20].

### 5.3. Asherman’s Syndrome

Asherman syndrome (AS) is a triad of pain, menstrual irregularities, and infertility caused by intrauterine scar tissue after the instrumentation of the uterus [69]. In 2023, Santamaria et al. investigated AS pathophysiology by comparing organoids from AS patient cells to healthy individuals [70]. The AS organoids had a loss of endometrial epithelium, altered epithelial differentiation signaling pathways (Wnt and Notch), and secretory leukocyte protease inhibitors during the implantation window. They also had changes in cell-to-cell communication and gene expression profiles, showing a dysfunctional pro-fibrotic, pro-inflammatory, and anti-angiogenic environment. Other studies focused on applying EOs in treating Asherman syndrome [71]. For instance, Hwang et al. demonstrated that transplanting endometrial tissue-derived organoids into a murine model of AS reduced the damaged structure of the AS endometrium by dramatically reducing fibrotic lesions and increasing cellular proliferation and vessel formation, eventually resulting in improved embryo implantation [16].

### 5.4. Endometrial Cancer

Endometrial cancer is one of the most common gynecological malignancies in women [72]. With endometrial cancer rates on the rise, there is an unmet need for biological models to study cancer formation, metastasis, and disease progression and recurrence. There are limited data on the molecular and cellular basis of endometrial cancers, especially in the rarer and more aggressive subtypes. This limitation is partly due to the lack of high-fidelity models that mimic the in vivo properties of human endometrial cancer. Fortunately, 3D organoids have emerged to mimic the microenvironment and cellular milieu more accurately than 2D models, and they also allow for a more in vivo-like representation of the endometrium [15,73]. Several studies have already used EOs to further investigate endometrial cancer. For example, Chen et al. used mouse models and organoids derived from endometrial cancer patients to study *SNORD14E* and how it affects those with endometrial cancer [74]. They discovered that *SNORD14E* was over-expressed in cancer tissues. Moreover, patients with high levels of the gene had worse prognosis without differences in distribution among the biomolecular classification subgroups of endometrial cancer. The authors also established that targeting SNORD14E with an antisense oligonucleotide (ASO) could be a promising treatment for endometrial cancer. Chen et al. created EOs models with mutations in *PTEN*, *PIK3A*, and *PI3KR1,* which are commonly associated with endometrioid adenocarcinoma. They observed that these co-mutations lead to faster tumor development [75]. Additionally, they also established a drug screening pipeline that tested 56 small molecule compounds, revealing varying responses among organoids. Certain organoids displayed drug resistance, while others, such as those treated with dacomitinib, showed promising responses. Other similar studies have highlighted the therapeutic potential of EOs in the novel treatments of endometrial cancer [76,77,78,79].

### 5.5. Endometrial Infection

Endometrial infections, particularly those caused by chronic or untreated infections, can lead to scarring and dysfunction of the endometrium, resulting in infertility [80]. In this context, the use of EEOs could help elucidate the mechanisms through which infection alters endometrial structure and function, providing insight into therapeutic approaches for managing infertility linked to infection.

Interestingly, Łaniewski et al. successfully simulated ascending infections of the female reproductive tract using EOs [81]. The authors infected the EOs with commensal and pathogenic bacteria such as *Lactobacillus crispatus, Gardnerella vaginalis,* and *Neisseria gonorrhoeae.* Of all the different bacteria strains, only *Neisseria gonorrhoeae* triggered a significant proinflammatory reaction and caused significant ultrastructural tissue changes. Łaniewski et al. were the first to describe gonococcal infection of epithelial cells in a 3D cell culture. Other studies later simulated infections of *Chlamydia trochamitis* using EOs [82]. Dolat et al. observed that *Chlamydia trochamitis* induced cytoskeletal reorganization and a change in the intracellular organelle positioning [83]. Additionally, the organoids can be co-cultured with neutrophils to reconstruct immune cell responses, revealing that specific effectors like CPAF and TepP limit neutrophil recruitment to infected organoids.

### 5.6. Environmental and Drug-Induced Toxicity

Emerging research highlights the toxic effects of BPA and certain neurotherapeutic drugs on endometrial function. BPA has been shown to disrupt epithelial–mesenchymal transition (EMT), Wnt/β-catenin signaling, and metabolic homeostasis, all of which are essential for maintaining endometrial integrity [8,84]. Additionally, some drugs can interfere with reproductive function at the molecular level. Notably, melatonin and resveratrol have been found to mitigate BPA-induced damage, restoring cellular integrity and reducing apoptosis [8]. These findings further emphasize the potential of EOs as a model for both environmental and drug-induced toxicology studies, providing valuable insights into reproductive health risks.

EOs are increasingly recognized as essential tools in reproductive toxicology research. Traditional preclinical models often fail to predict the reproductive side effects of neurotherapeutic drugs, leading to unforeseen complications in clinical trials [84]. Recent advancements highlight how organoids can serve as alternative models for drug safety testing, helping to assess reproductive toxicity early in drug development. The integration of high-throughput drug screening with organoid platforms, alongside environmental exposure studies, may further enhance personalized medicine approaches in reproductive health.

## 6. Impact of Organoids on Assisted Reproductive Technologies (ARTs) and Biomarker Development

Infertility is a huge problem in today’s societies and has a significant social and psychological impact on women. Treatment is needed—from medical and surgical interventions to assisted reproductive technologies (ARTs)—depending on the cause [32]. Despite the many ways to administer external estrogen and progesterone to prepare the endometrium in women undergoing in vitro fertilization (IVF)—one of the ART methods available today—achieving a clinical pregnancy is not guaranteed. There is ongoing debate about the type, dosage, and timing of these hormonal supplements [85]. For example, high doses of estrogen are essential during the window of implantation, but high doses of estrogen have been associated with implantation failures in mice and increased risk of ovarian hyperstimulation syndrome. On the other hand, high concentrations of progesterone used to treat luteal phase deficiency negatively impact uterine receptivity and decidualization, as shown in mice in vivo and immortalized human endometrial stromal cells in vitro [86,87,88]. Another major cause of infertility is the inability of human embryos to implant successfully. This is highlighted by frequent early pregnancy losses and the low success rate of IVF, which is 31% per fresh embryo transfer, as reported by 2022 data from the Human Fertilization and Embryology Authority (HFEA) [89]. Modeling implantation is key to understanding the interactions between the embryo and the mother during early pregnancy. Despite the progress in IVF technology, understanding the implantation process is still a major limitation of IVF treatments [90]. Implantation cannot be studied directly in the human body, so scientists rely on animal models and cell culture methods. However, recent developments in 3D modeling, especially with organoids, offer new avenues to explore previously inaccessible aspects of human reproduction [91]. EOs can be generated from biopsies obtained from infertile women undergoing IVF treatment—using either fresh or frozen samples—and exposed to varying concentrations of estrogen and progesterone, to evaluate their effects on cellular proliferation and differentiation [64]. The expression of estrogen receptor (ER) and progesterone receptor (PR) can also be assessed in these organoid cultures to see if the endometrium can achieve a receptive state in response to progesterone [32]. Various in vitro models have been developed to simulate implantation, which is a key to improving IVF success rates, including primary endometrial epithelial cultures, immortalized epithelial cell lines, and cell lines such as Ishikawa, low-receptivity HEC-1-A, high-receptivity RL95-2 and ECC-1 derived from endometrial carcinoma. Decidualization models use the hormonal stimulation of ESCs, while embryo surrogate models use trophoblastic cell lines and spheroids. More complex assays like Transwell are also used. Although these systems mainly focus on the attachment or invasion of embryo surrogates to a 2D monolayer, they are useful for modeling specific stages of implantation and screening factors that influence embryo adhesion and invasion [92]. Rawlings et al. created an endometrial assembloid to study embryo implantation and decidual senescence, defined as a metabolically active state without cell division [91]. In their model, they embedded a human embryo into the assembloid and added dasatinib, a tyrosine kinase inhibitor that gets rid of senescent decidual cells. Senescence is important to prevent miscarriage and implantation failures, but the removal of these cells, by dasatinib, trapped the embryo in a non-progressive decidual matrix and prevented implantation. However, the model is limited as it does not resemble the real endometrium as it lacks the lining epithelium and immune components. To improve the still-imperfect in vitro conditions for gamete preparation and embryo culture during in vitro fertilization (IVF), researchers created a human fallopian tube organoid from adult stem cells to culture spermatozoa in the apical compartment. Human fallopian tubes are the site of key events for a successful pregnancy, and modeling them could improve IVF outcomes. Their model differentiated into fallopian tube cells, as confirmed by multiple analyses, and worked for sperm culture.

Sperm vitality in the human fallopian tube (HFT) organoids was similar to commercial sperm media. Notably, sperm motility was higher in the HFT organoids than in all other conditions and remained higher even after 96 h compared to all other tested environments [93]. In another study, Barry et al. showed that a biphasic (5–2%) O_2_ concentration during preimplantation embryo culture improves blastulation and live birth rates in IVF [94]. Their study shows that the transcriptomic analysis of trophoblast organoids cultured in different O_2_ concentrations can be used to mimic in vivo conditions and improve our understanding of embryo development in ARTs. HIF1a was upregulated in biphasic conditions and not in monophasic conditions, and organoid models can provide valuable insights into embryo angiogenesis and vascularization and improve ART outcomes.

Repeated Implantation Failure (RIF) is a clinical condition diagnosed in the context of ARTs and is often associated with endometrial receptivity and endometrium–embryo interaction [65,95]. A previous study on endometrial transcriptomes from whole-tissue biopsies showed numerous differentially expressed genes (DEGs) in the endometrium of women with RIF compared to fertile controls [96]. This means there are differences in endometrial function between the two groups that impact fertility outcomes. Hormone imbalance can affect the timing of endometrial receptivity, which is critical for the synchronization of successful implantation. One study showed that 2D epithelial monolayers treated with estradiol (E_2_), progesterone (P_4_), 8- Br-cAMP(C), and XAV939 (X)—shortly EPCX—expressed more receptivity genes and had higher blastoid adhesion rates than controls. Blastoids are structures derived from stem cells that resemble the blastocyst stage of human embryos. This setup allowed for the precise measurement of functional implantation failure and showed significant trophoblast cell migration. These results open up possibilities for the further study of hormonal impact on implantation failure, but the model is limited by its simplicity (lacking stromal, endothelial, and immune cells) [95]. Clinical studies have shown that intrauterine insemination of seminal plasma (SP) during assisted reproductive technologies (ARTs) can improve implantation and pregnancy rates [97]. The exact molecular mechanisms by which SP works is not fully understood. SP contains TGF-b1 or IL-8, which can condition the uterus to be more receptive to pregnancy [98]. SP also induces notable transcriptional changes in 2D endometrial epithelial cells. Recently developed 3D human EOs, which more closely mimic in vivo conditions, can provide a better understanding of how the endometrium interacts with SP. This was tested in a study where endometrial tissue samples from fertile and sub-fertile women (defined as those not achieving pregnancy after at least a year of regular unprotected intercourse) were used to create endometrial epithelial organoids [99]. SP from fertile men was collected, pooled, and used in the experiment. The organoids were grown in a complex medium enriched with 17β-estradiol (E_2_) and incubated with or without a small amount of SP for six hours. The study showed significant variation between samples from different donors. This variation was attributed more to individual differences than to fertility status or SP treatment. This shows how ARTs can advance by using organoids to study how seminal plasma affects endometrial receptivity. Results can lead to personalized treatment and improved implantation strategies and increase ART success rates.

Alternatively, organoids can be derived from non-invasive methods such as menstrual flow-derived organoids, as biopsies of the endometrial tissue may be considered difficult or non-feasible due to ethical considerations. Davies et al. demonstrated that both sets of organoids exhibit identical transcriptome signatures, derivation efficiency, and proliferation rates [18]. Such findings hold significant potential for enhancing assisted reproductive technologies by enabling non-invasive investigations and tailored treatments for reproductive disorders, including failed implantation following IVF and recurrent miscarriage.

## 7. Biomarker Development

A biomarker is a measurable indicator of cellular or organismal state at a given time. Biomarkers are essential for understanding the link between environmental chemicals and human disease, to diagnose, track, and predict disease risk [100]. EOs derived from human biopsies are a great platform for biomarker development in reproductive health (Table 1). They can be grown long-term, are genetically stable, and can be frozen [101,102]. By exposing them to hormones like estrogen and medroxyprogesterone acetate, changes in gene expression can be simulated, like the endometrial cycle. This is supported by markers like FOXA2 and changes in steroid hormone receptor expression. Techniques like real-time qPCR and bulk RNA sequencing confirm that hormone treatment induces gene expression in a way that mimicked the proliferative and secretory phases of the endometrium [103].

Biomarkers can serve as prognostic tools for the pathologic classification of endometrial carcinoma, which remains inconsistent [104]. To address this, Cochrane et al. and others employed single-cell sequencing to study organoid model systems derived from normal endometrial tissue to identify new markers for endometrial ciliated or secretory cells [113]. Secretory cell marker (MPST) and ciliated cell markers (FAM92B, WDR16, and DYDC2) were validated using immunohistochemistry on organoids and tissue sections. Furthermore, single-cell sequencing of endometrial and ovarian tumors revealed secretory-like and ciliated-like tumor cells. Notably, the expression of ciliated cell markers (DYDC2, CTH, FOXJ1, and p73) and the secretory marker MPST found in endometrial tumors correlated with better survival outcomes, suggesting their utility in stratifying endometrial carcinoma by aggressiveness.

Glycodelin-A (GdA), a glycoprotein from the lipocalin superfamily, plays a role in reproduction and fetal–maternal immune tolerance due to its unique glycan structures [114]. Recent studies showed that both the expression and glycoform of GdA are changed in the eutopic endometrium of women with endometriosis [105]. Interestingly, organoids derived from these patients replicate this altered expression while maintaining morphological similarity to a healthy endometrium. This makes organoids a valuable tool for studying implantation biology and for developing biomarkers to aid in the diagnosis and treatment of gynecologic disorders [115].

## 8. Current Limitations and Future Insights of Endometrial Organoids

EOs have emerged as a powerful tool for studying the human endometrium, providing new insights into endometrial biology, disease mechanisms, and therapeutic strategies. However, like any model system, they come with inherent limitations that must be addressed to fully realize their potential.

### 8.1. Technological Limitations

Currently, Matrigel, which was isolated from Engelbreth–Holm–Swarm mouse sarcoma, is used as the primary extracellular matrix in the EO culture [116]. For animal-derived and ill-defined traits, it may not fully mirror the human endometrial environment, despite its effectiveness in encouraging organoid growth and self-organization. Several artificial and chemically defined hydrogels, extracellular matrix hydrogels made from decellularized tissues, and microstructured collagen scaffolds have been developed for 3D cell culture and organoid cultures to overcome these limitations [106,107]. Furthermore, endometrial epithelial and stromal cells have been co-cultured using collagen scaffolds and stromal cell-induced scaffolds using agarose 3D Petri dishes, suggesting that alternative types of culture materials—rather than Matrigel—are equally viable in the EO culture [108,109].

Recent innovations in microfluidic systems and synthetic extracellular matrices (ECMs) have significantly improved EO models [20]. These systems allow for precise control over hormonal fluctuations, nutrient exchange, and cellular interactions, bringing organoid models closer to clinical applications in fertility research.

Because EOs have a basal out/apical phenotype, they are not completely identical to the endometrium in vivo. This makes it difficult to explore their surface markers in binding assays. Furthermore, transfecting nucleic acids into cells is challenging due to the organoid epithelium tight junctions. Simintiras et al. discovered that certain metabolites were specific to extra-organoid fluid, which was the result of the organoid system’s asymmetrical apical and basolateral secretion [110]. Apical proteins in organoids could be studied in this scenario by microinjection [83]. To overcome these difficulties, an organoid-specific transfection methodology should be created, and a matrix gel-independent suspension culture system should be utilized for reverse polarity [111].

It is unclear how organoids interact with other tissues, mostly because of the polarity and the question of whether co-cultured cells, tissues, and organoids would be affected by Matrigel or EO media. The establishment of corresponding co-culture systems is necessary to replicate real in vivo conditions. Moreover, microfluidic technologies may be used to solve the challenging in vitro alignment of various biological systems [117]. For instance, a microfluidic device was developed to replicate the characteristics of the human menstrual cycle in 28 days using various tissues from the female reproductive tract and sex hormones from ovarian follicles [118]. To investigate the communication between the human ovary and endometrium, an organ-on-a-chip system was also created [112]. Chip models and organoids can be used to replace 2D cell cultures and tissue explants, improving the biological relevance to the original tissue.

### 8.2. Biological Constraints

There are technical challenges in the establishment and maintenance of EOs because of the lack of cellular heterogenicity and the limited cell types. Currently, EOs can only be used to study epithelial cells rather than other cell types such as stromal and immune cells which also play an important role in disease development [118]. Co-culturing stromal and endometrial epithelial cells has been accomplished in several experiments, as previously mentioned. Instead of using Matrigel, the EOs were cultivated in novel ECM types. While in these models stromal cells were cultured using agarose-based 3D Petri dishes to generate their own ECM, others used preformed 3D porous collagen scaffolds as the ECM substrate [36,107]. Thus, it can be seen that co-culture techniques and the investigation of new cultural ECMs benefited from one another. These co-culture models, however, lack maturity and persuasiveness because it was not determined whether they could be long-term or phenotypically and genotypically stable. Additionally, immune system anomalies and vascular dysfunction are intimately linked to endometrial and pregnancy disorders. Pregnancy, for instance, is facilitated by communication between the epithelium, decidualized stromal cells, resident immune cells, vasculature, and placenta trophoblast; any form of cell dysfunction would impact the outcome. The function of immune cells and the vasculature must be taken into account to investigate the pathogenesis. Using agarose 3D Petri dishes to study scaffold-free EOs, no immune cells were virtually present based on the detection of typical surface markers. Additionally, there is no a report on the vasculature’s function in EOs yet. As a result, EOs should enhance the process for co-culturing stromal and epithelial cells while working to investigate the technology for co-culturing immune and endothelial cells [100].

### 8.3. Translational Barriers in Endometrial Organoid Transplantation (EOT)

The findings of this review, which synthesize a wide range of preclinical evidence in favor of EOT, are primarily descriptive reviews of previous research rather than critical analyses. To put these developments into perspective, a more thorough analysis of methodological limitations and translational barriers is necessary. To generate organoids, for example, several research studies use heterogeneous protocols that vary in scaffold materials, transplantation methods, and cell sources (e.g., primary cells vs. stem cells) [47,48,53]. This discrepancy makes direct comparisons more difficult to determine the ideal settings for clinical use. Additionally, whereas preclinical models show encouraging structural and functional recovery, they frequently fail to account for species-specific variations in endometrial physiology, which calls into question whether results can be extrapolated to humans.

Clinical translation still faces difficulties in spite of these developments. Human-specific data is still scarce, and the majority of studies use rodent models. Santamaria et al. emphasized the need for patient-specific optimization by highlighting the transcriptome differences between organoids produced from patients with Asherman’s syndrome and healthy controls [70]. Standardization is complicated by protocol variability, including time of transplantation, culture conditions (Matrigel vs. fibrin hydrogels), and organoid sources (primary cells vs. stem cells). Although there are currently no negative results from trials, safety concerns, such as the tumorigenic potential of stem cell-derived organoids, necessitate further long-term research [55]. Another obstacle is scalability because it still requires considerable resources to produce clinical-grade organoids and scaffolds.

Future research should focus on mechanistic studies to elucidate organoid–host interactions, standardized techniques to improve reproducibility, and cooperative frameworks to tackle ethical and regulatory issues in order to close these gaps. Prioritizing human trials to assess safety and effectiveness in conditions like AS or thin endometrium is essential for future advancement. Reproducibility will be improved by standardizing procedures for organoid culture, scaffold composition, and transplantation timing. While mechanistic research clarifying mitochondrial transfer and immunomodulatory pathways may improve therapeutic targeting, autologous techniques employing patient-derived cells may reduce the danger of immunological rejection [54].

EOT is a revolutionary approach to treating infertility caused by endometrial malfunction. For patients with few other therapy options, EOT presents a viable solution by promoting embryo implantation, improving vascularization, and repairing tissue architecture. Despite the tremendous preclinical promise of EOT, the transition from lab to bedside runs the risk of halting without a thorough review.

### 8.4. Ethical Considerations

The use of animal-derived materials such as Matrigel raises ethical concerns related to animal welfare, reproducibility, and translational relevance. As Matrigel is sourced from mouse sarcoma, its continued use may limit the clinical applicability of findings and conflicts with efforts to develop fully defined, xeno-free culture systems. Moreover, future applications of EOs in personalized medicine, reproductive health, and genetic manipulation will require careful ethical evaluation, particularly regarding consent, data privacy, and potential off-target effects in gene-editing approaches. Despite the present limitations of EOs, the current results are promising and warrant further experiments and studies in the field. Eos.

## 9. Conclusions

EOs have served as a physiological model, resembling in vivo tissue, for studying endometrial receptivity and implantation. Their ability to respond to hormonal stimuli and mimic the cyclic hormonal changes of the endometrium has made them of great value for studying implantation failures and pregnancy loss. Moreover, they hold a promising future in restoring function in patients with infertility due to endometrial dysregulations. Taking into account the various limitations of current models, future research should focus on integrating microfluidic systems, co-culturing with immune and stromal cells, and improving transplantation techniques to optimize their therapeutic potential. Their therapeutic potential may be further enhanced by developments in bioengineering and personalized medicine, opening the door to patient-specific therapies.

## Figures and Tables

**Table 1:** This table summarizes the studies included in which organoids were utilized successfully and aids reader(s) in navigating through cited works. IO: Intestinal Organoid; LO: Liver Organoid; BO: Brain Organoid; EO: Endometrial Organoid; PDOs: Patient-derived Organoids; EGO: Endometrial Gland Organoid; HO: Human Organoid; UO: Urothelial Organoid; PCOS: Polycystic Ovarian Syndrome; UEM: Uterus Extracellular Matrix; hPSC-MuDO: Human pluripotent stem cell derived Müllerian duct-like organoid; EnO: Endometriosis Organoid; MDOs: Mice-derived Organoids; PDXOs: Patient-derived Xenograft Organoids; GC-PDOs: Gynecologic Cancer Patient-derived Organoids; EC-PDOs: Endometrial Cancer Patient-derived Organoids; HFTOs: Human Fallopian Tube Organoids; TO: Trophoblast Organoid; EndodO: Endodermal Organoid; GIO: Gastrointestinal Organoid.

Study	Organoid Type	Research Focus	Key Findings	Impact	Published in
Sato T, et al. (2009) [2]	IO	Organoid formation	Demonstrated that single Lgr5+ intestinal stem cells can generate self-organizing crypt-villus structures	Pioneering study that launched the modern era of organoid research	*Nature*
Jabri A, et al. (2024) [4]	LO	Cancer modeling	Reviewed liver cancer organoid development, emphasizing their value for studying hepatocellular carcinoma	Highlights organoid utility in liver cancer research and therapeutic discovery	*Bioengineering (Basel)*
Smirnova L, et al. (2024) [5]	BO	Translational neuroscience	Reviewed applications of brain organoids in modeling neurological diseases and drug development	Highlights brain organoids as powerful tools for brain research and personalized medicine	*Adv Healthc Mater*
Guo J, et al. (2023) [6]	EO	Endometrial receptivity	Explores how endometrial organoids can be used to study human endometrial receptivity and factors influencing implantation	Contributes to reproductive health by advancing our understanding of implantation and fertility	*Front Endocrinol (Lausanne)*
Abady, M. M., et al. (2024) [8]	EO	Toxicology, Reproductive health	Demonstrated the protective effects of melatonin and resveratrol in BPA-induced endometrial toxicity	Advances our understanding of environmental toxins and reproductive health	*Reproductive Toxicology*
Turco MY, et al. (2017) [11]	EO	Endometrial culture and hormonal regulation	Developed long-term, hormone-responsive organoid cultures of human endometrium, crucial for studying menstrual cycles and implantation	Provides a model for studying endometrial diseases and infertility	*Nat Cell Biol*
Juárez-Barber E, et al. (2023) [13]	EO	Embryo implantation and pregnancy	Identified miRNAs in extracellular vesicles that are involved in embryo implantation and pregnancy, highlighting the potential of organoids for reproductive research	Provides insights into the molecular mechanisms of embryo implantation and pregnancy using endometrial organoids	*Reproductive Biomedicine Online*
Boretto M, et al. (2019) [15]	EO	Disease modeling, Drug screening	Patient-derived endometrial organoids were created to capture clinical heterogeneity in endometrial diseases and were used for drug screening	Advances in personalized medicine and drug screening for endometrial diseases	*Nat Cell Biol*
Hwang SY, et al. (2024) [16]	EO	Uterine repair	Identified endometrial organoids as a source of functional mitochondria, highlighting their potential in uterine repair and regenerative medicine	Opens new avenues for uterine tissue repair, offering a potential therapeutic strategy for infertility related to uterine dysfunction	*Theranostics*
Tamura H, et al. (2018) [17]	PDOs	Evaluation of anticancer agents	Patient-derived tumor organoids were shown to closely resemble the source tissues and can be used to evaluate anticancer agents	Provides a valuable model for personalized cancer therapy by testing drug efficacy on patient-derived organoids	*Oncol Rep*
Cindrova-Davies T, et al. (2021) [18]	EO	Non-invasive sources of EO	The study demonstrated that menstrual flow can be a viable source for generating endometrial organoids	Offers a non-invasive method for obtaining endometrial tissue, which could improve research on endometrial diseases	*Commun Biol*
Marinić M, et al. (2020) [19]	EGO	Derivation of EGO	Successful derivation of organoids from the term placenta, offering a model to study endometrial function	Provides a new in vitro model for investigating endometrial biology, useful for understanding endometrial and placental interactions	*Placenta*
Saadeldin IM, et al. (2024) [20]	HO	Maternal–embryonic interaction	Reviewed 3D organoid models utilized to study the interactions between maternal and embryonic cells in vitro.	Advances understanding of maternal–embryonic communication, providing insights into fertility and implantation.	*J Vet Sci*
Boretto M, et al. (2017) [21]	EO	Reproductive biology, Endometrial physiology	Developed human and mouse endometrial organoids, demonstrating endometrial epithelium physiology and long-term expandability	Provides a model for studying endometrial function and related diseases, with potential applications in fertility research	*Development*
Santos CP, et al. (2019) [23]	UO	Regenerative medicine	Urothelial organoids display Notch-dependent differentiation capacity, providing insights into urothelial tissue development and regenerative therapies	Advances the understanding of urothelial biology and organoid-based models for regenerative medicine and drug testing	*Nat Commun*
Wang X, et al. (2020) [24]	LO	Liver disease modeling	Successful generation of liver bipotential organoids that can differentiate into both hepatocytes and cholangiocytes, providing a model for liver disease modeling and regenerative medicine	Provides a novel organoid model for liver disease research, drug testing, and potential therapeutic applications	*J Mol Cell Biol*
Haider S, et al. (2019) [25]	EO	Endometrial diseases	Estrogen signaling was found to drive ciliogenesis in endometrial organoids, shedding light on the hormonal regulation of cilia formation in the endometrium	Advances understanding of estrogen’s role in the endometrium, with potential implications for fertility and endometrial diseases	*Endocrinology*
Bates RC, et al. (2003) [26]	HO	Epithelial tumors	TNF-α was found to induce EMT in human colonic organoids, a key process in cancer metastasis	Provides insight into the mechanisms of metastasis and the role of inflammation in promoting EMT, relevant for cancer research	*Mol Biol Cell*
Nikolakopoulou K, et al. (2021) [32]	EO	Infertility and reproductive health	Focused on how endometrial organoids are used to investigate infertility, with an emphasis on embryo implantation	Provides insights into the use of organoids for studying fertility issues and endometrial function	*Reproduction*
Garcia-Alonso L, et al. (2021) [33]	EO	Infertility	Mapping of the endometrial dynamics to understand its role in fertility and diseases	Provides valuable insights into endometrial biology and could improve disease modeling and therapeutic strategies	*Nat Genet*
Wiwatpanit T, et al. (2020) [36]	EO	PCOS	Demonstrated that scaffold-free endometrial organoids respond to excess androgens, a hallmark of PCOS, revealing potential mechanisms of disease	Contributes to understanding the pathophysiology of PCOS and could inform therapeutic strategies for managing androgen excess and related reproductive issues	*J Clin Endocrinol Metab*
Shibata S, et al. (2024) [37]	EO	Embryo–endometrial interface	Developed a model to study the embryo–endometrial interface, recapitulating human embryo implantation in vitro	Provides new insights into the molecular interactions during embryo implantation, offering potential for fertility treatments	*Sci Adv*
Cha E, et al. (2024) [47]	EO	Endometrial diseases	UEM supports endometrial organoid development more effectively than Matrigel; decorin in UEM enhances Wnt7a expression; UEM improves epithelial regeneration and pregnancy rates in vivo	Provides a biomimetic scaffold that enhances organoid development and has potential therapeutic applications in endometrial regeneration	*Advanced Functional Materials*
Dai Y, et al. (2024) [48]	EO	Organoid development	Successfully engineered vascularized endometrial organoids that integrate with host tissue, promoting regeneration and repair in vivo	Demonstrates the potential of vascularized organoids in endometrial tissue engineering and regenerative medicine	*Human Reproduction*
Gong L, et al. (2022) [49]	hPSC-MuDO	Regenerative medicine	Successfully generated hPSC-derived Müllerian duct-like organoids capable of differentiating into both epithelial and stromal lineages, leading to the regeneration of full-thickness endometrial tissue in vivo	Demonstrates the potential of hPSC-derived organoids in endometrial tissue engineering and regenerative medicine	*npj Regenerative Medicine*
He et al., (2022) [50]	MDO	Organoid development	SSEA-1^+^ cells self-organized into organoid structures with long-term expansion capacity; transplantation in a rat IUA model reduced fibrosis and facilitated endometrial regeneration	Demonstrates the potential of stem cell-derived organoids in endometrial regeneration and treatment of IUA	*Cell and Bioscience*
Zhang H, et al. (2022) [51]	EO	Organoid transplantation	Organoid transplantation improved endometrial repair and reproductive prognosis in mice	Highlights potential for organoid-based therapies in endometrial regeneration and fertility restoration	*Int J Biol Sci*
Jiang X, et al. (2021) [71]	EO	Endometrial diseases	Successful regeneration of endometrial tissue using stem cell-derived organoids and 3D Matrigel in Asherman syndrome	Demonstrates the potential of organoids in treating endometrial damage and infertility	*Bioact Mater*
Wiweko B. (2023) [53]	EO	Infertility, Regenerative medicine	Intrauterine PRP shows promise in improving implantation rates; 3D acellular scaffolds with autologous endometrial cells demonstrate potential in regenerating endometrial tissue in cases unresponsive to conventional therapies	Highlights innovative approaches combining intrauterine therapies with tissue engineering for endometrial regeneration	*Fertility and Reproduction*
Esfandiari F, et al. (2021) [62]	EnO	Endometrial diseases	Identified DNA methylation patterns in HOX genes and their cofactors specific to endometriosis	Provides insights into epigenetic regulation in endometriosis, advancing understanding of the disease at the molecular level	*Fertil Steril*
Esfandiari F, et al. (2021) [63]	EnO	Endometrial diseases	Found that progesterone signaling was disturbed in a preclinical model of advanced endometriosis	Provides valuable insights into the hormonal dysregulation in endometriosis, advancing therapeutic targets for treatment	*Reprod Biomed Online*
Bui BN, et al. (2020) [64]	EO	Endometrial diseases	Demonstrated the successful establishment of organoids from cryopreserved endometrial tissue of infertile women, showing organoid culture feasibility	Provides a reliable model for fertility research and endometrial disease studies	*Reprod Biomed Online*
Bui BN, et al. (2024) [67]	EO	Infertility	Enrichment of cell cycle pathways in organoids compared to fertile women	Advances understanding of progesterone treatment in fertility	*J Assist Reprod Genet*
Hibaoui Y, et al. (2020) [73]	EO	Endometrial development and disease	Discussed the creation of organoid models to study endometrial development and diseases, focusing on their potential applications in understanding endometrial pathology	Provides valuable insight into the potential of organoid models in studying endometrial diseases	*Front Cell Dev Biol*
Chen X, et al. (2023) [74]	PDOs	Endometrial cancer, therapeutic target identification	Identified an ASO targeting SNORD14E for endometrial cancer, reducing FOXM1 expression and β-catenin nuclear accumulation.	Contributes to the development of new therapeutic strategies for endometrial cancer	*J Exp Clin Cancer Res*
Chen J, et al. (2023) [75]	MDOs	Endometrial cancer, genetic drivers, cancer model	Developed a new model for endometrial cancer in mice, revealing the roles of genetic drivers and their treatment susceptibilities	Provides insights into the genetic underpinnings of endometrial cancer and aids in the development of targeted therapies	*Adv Sci (Weinh)*
Maru Y, et al. (2019) [76]	PDOs	Gynecologic tumors, preclinical model	Efficient use of PDOs as models for gynecologic tumors, allowing for accurate replication of patient cancer characteristics and treatment testing	Provides a platform for personalized treatment strategies, enhancing understanding of gynecologic cancer biology	*Gynecol Oncol*
Sengal AT, et al. (2023) [77]	PDXOs	Endometrial cancer, FGFR2c isoform expression, drug sensitivity	PDXOs with FGFR2c isoform expression are sensitive to FGFR inhibition, indicating the potential for targeted therapies.	Demonstrates the use of organoids for personalized medicine and preclinical drug testing in endometrial cancer.	*NPJ Precis Oncol*
Bi J, et al. (2021) [78]	GC-PDOs	Gynecologic cancer modeling and drug sensitivity testing	Successfully cultured patient-derived organoids from gynecologic cancers for disease modeling and drug sensitivity testing.	Supports the development of personalized treatment approaches for gynecologic cancers.	*Cancers (Basel)*
Girda E, et al. (2017) [79]	EC-PDOs	Exploring drug sensitivity testing	Demonstrated feasibility of using organoids for drug sensitivity testing	Enables personalized cancer treatment approaches	*Int J Gynecol Cancer*
Bishop RC, et al. (2020) [81]	Murine EO	Modeling Chlamydia infection in the endometrium	Successfully used murine endometrial organoids to study Chlamydia infection dynamics	Provides a valuable model for investigating reproductive tract infections and potential treatments	*Front Cell Infect Microbiol*
Dolat L, et al. (2021) [82]	EO	Interactions between Chlamydia and epithelial/immune cells	Successfully models Chlamydia interactions with epithelial and immune cells in the endometrium	Provides a new model for studying immune responses and bacterial infections in the reproductive tract	*J Cell Sc*
Abady MM, et al. (2024) [83]	HO	Drug toxicity	Organoid models can be used to assess the reproductive toxicity of neurogenic/neuroprotective drugs, an alternative to animal models	Provides evidence that organoid models can offer more accurate insights into drug toxicity, particularly for reproductive safety	*Front Pharmacol*
Rawlings TM, et al. (2021) [90]	EO	Endometrial implantation	Focused on using organoids to model endometrial implantation processes and other related functions	Highlights the potential of organoid models in studying endometrial biology and improving reproductive health therapies	*Reproduction and Fertility*
Kleinová M, et al. (2024) [91]	EO	Generation, implantation modeling, and clinical perspectives	Explores the potential of endometrial organoids and assembloids for studying reproduction, including modeling implantation processes	Provides insight into the future clinical applications of organoid models in reproductive health	*Front Cell Dev Biol*
Gatimel N, et al. (2024) [92]	HFTOs	Sperm motility and organoid environment	Demonstrates that human fallopian tube organoids support sperm motility, which could enhance understanding of fertility and reproduction	Highlights the potential of organoid models to mimic reproductive tract conditions and provide a better understanding of fertility	*Human Reproduction*
Barry F, et al. (2024) [93]	TO	Embryo implantation and pregnancy	Developed trophoblast organoids to study the effects of oxygen concentration on preimplantation embryo culture, offering insights into the early stages of pregnancy	Provides a novel 3D model to explore trophoblast function and the impact of oxygen levels on embryo development, which is crucial for improving in vitro fertilization techniques	*Human Reproduction*
Van den Berg J, et al. (2024) [98]	EO	Endometrial receptivity	Seminal plasma exposure altered the transcriptomic profile of endometrial organoids, revealing differences based on fertility status	Provides insight into how seminal plasma may modulate endometrial receptivity and fertility, using a physiologically relevant organoid model	*Human Reproduction*
Deane JA, et al. (2017) [100]	EO	Endometrial research and personalized medicine	The study highlights the potential of endometrial organoids as in vitro models for studying endometrial diseases and personalized medicine.	Demonstrates the utility of endometrial organoids for disease modeling and patient-specific drug testing.	*Biol Reprod*
Gu ZY, et al. (2020) [101]	EO	Endometrial diseases	This study introduces endometrial organoids as a novel model for investigating endometrial diseases, providing insights into disease mechanisms and potential therapeutic strategies	Supports the potential of endometrial organoids as a reliable in vitro model for advancing research and treatment strategies for endometrial diseases	*Biol Reprod*
Fitzgerald HC, et al. (2019) [102]	EO	Self-renewing endometrial epithelial organoids in human uterine research	The study demonstrates the successful establishment of self-renewing endometrial epithelial organoids from the human uterus, which can be used to investigate endometrial biology and disease mechanisms	Highlights the potential of human endometrial organoids in advancing the understanding of endometrial diseases and in personalized medicine	*PNAS*
Cochrane DR, et al. (2020) [104]	EO	Cell type biomarkers	Identified novel cell type markers and cryptic differentiation patterns in primary tumors using normal endometrial-derived organoids	Enhances understanding of cellular heterogeneity in endometrial tissues and can aid in better characterization of endometrial cancers	*J Pathol*
Luddi A, et al. (2020) [105]	EO	Endometrium-embryo cross-talk at the implantation site	Human endometrial organoids model the interactions between the endometrium and embryo at the implantation site	Provides a novel in vitro tool for studying implantation processes in fertility research	*Cells*
Giobbe GG, et al. (2019) [106]	EndodO	Organoid development	Demonstrated that tissue-specific ECM hydrogels can support long-term growth and maintenance of endodermal organoids	Offers a biocompatible, physiologically relevant scaffold to improve organoid culture fidelity	*Nature Communications*
Murphy AR, et al. (2019) [107]	EO	Organoid development	Successfully established and visualized a reproducible method for creating endometrial organoids that include both epithelial and stromal components	Provides a reliable and accessible model for studying human endometrial physiology and pathophysiology	*Journal of Visualized Experiments*
Abbas Y, et al. (2020) [108]	EO	Organoid development	Successfully generated a multicellular human endometrial model incorporating stromal and epithelial cells, recapitulating structural and functional features	Enhances the ability to study implantation, regeneration, and menstrual cycle processes in vitro	*Interface Focus*
Simintiras CA, et al. (2021) [109]	EO	Organoid development	Identified and profiled metabolites secreted by endometrial organoids, revealing active metabolic signaling relevant to implantation and endometrial function	Offers insights into endometrial physiology and potential biomarkers for reproductive health	*PNAS*
Co JY, et al. (2021) [110]	GIO	Organoid development	Demonstrated a method to control organoid polarity, enabling luminal access and better modeling of host–pathogen interactions	Facilitates advanced studies of gastrointestinal infections and epithelial function in a physiologically relevant model	*Nature Protocols*
Li JL, et al. (2022) [111]	EO	Organoid development	Developed endometrial organoids to model key features of the human endometrium, useful for understanding menstrual cycle dynamics and related disorders	Provides a powerful model for studying endometrial biology and endometrial-related diseases in vitro	*Reproductive and Developmental Medicine*
Esfandiari F, et al., (2022) [112]	EnO	Prospects and challenges of using endometriosis organoids	Discussed the potential applications and challenges of generating endometriosis organoids for studying the disease and testing treatments	Highlights the promise of organoids in advancing research on endometriosis, while also addressing technical challenges in their use for therapeutic development	*Reprod Biomed Online*

## Abbreviations

The following abbreviations are used in this manuscript: **2D**—Two dimensional, **3D**—Three dimensional, **8-Br-cAMP**—8-Bromoadenosine 3′,5′-cyclic monophosphate, **A83-01**—TGF-β inhibitor, **ARTs**—Assisted Reproductive Technologies, **AS**—Asherman’s Syndrome, **AXIN2**—Axis Inhibition Protein 2, **BMP**—Bone Morphogenetic Protein, **BO**—Brain Organoids, **BPA**—Bisphenol A, **CDH1**—Cadherin 1 (E-cadherin), **CPA3**—Carboxypeptidase A3, **CPAF**—Chlamydial Protease-Like Activity Factor, **CTH**—Cystathionine Gamma-Lyase, **DMEM/F12**—Dulbecco’s Modified Eagle Medium/Nutrient Mixture F-12, **DPP4**—Dipeptidyl Peptidase 4, **DYDC2**—DPY30 Domain Containing, **ECM**—Extracellular Matrix, **EGF**—Epidermal Growth Factor, **EMT**—Epithelial-Mesenchymal Transition, **EnO**—Endometriosis Organoids, **EPCAM**—Epithelial Cell Adhesion Molecule, **EPCX**—Estradiol (E2), Progesterone (P4), 8-Br-cAMP (C), XAV939 (X), **EOs**—Endometrial Organoids, **EOT**—Endometrial Organoid Transplantation, **ER**—Estrogen Receptor, **ER-α**—Estrogen Receptor alpha, **ESCs**—Endometrial Stromal Cells, **FAM92B**—Family With Sequence Similarity 92 Member B, **FGF10**—Fibroblast Growth Factor 10, **FOXJ1**—Forkhead Box J1, **FOXA2**—Forkhead Box A2, **GC-PDOs**—Gynecologic Cancer Patient-Derived Organoids, **GdA**—Glycodelin-A, **GPER**—G Protein-Coupled Estrogen Receptor, **HFT**—Human Fallopian Tube, **HGF**—Hepatocyte Growth Factor, **HIF1a**—Hypoxia-Inducible Factor 1-alpha, **HOX**—Homeobox genes, **hPSCs**—Human Pluripotent Stem Cells, **hPSC-MuDO**—hPSC-derived Müllerian Duct-like Organoids, **HSD17B2**—Hydroxysteroid 17-Beta Dehydrogenase 2, **HSPA9**—Heat Shock Protein A9, **IUA**—Intrauterine Adhesions, **IVF**—In Vitro Fertilization, **LH**—Luteinizing Hormone, **LGR5**—Leucine-rich repeat-containing G-protein coupled Receptor 5, **LIF**—Leukemia Inhibitory Factor, **Liberase**—Enzyme blend for tissue dissociation, **LRIG1**—Leucine-Rich Repeats and Immunoglobulin-Like Domains 1, **MAPK**—Mitogen-Activated Protein Kinase, **MDLCs**—Müllerian Duct-Like Cells, **MPST**—Mercaptopyruvate Sulfurtransferase, **MUC1**—Mucin 1, **Noggin**—BMP signaling inhibitor, **PAEP**—Progestogen-Associated Endometrial Protein, **PAX8**—Paired Box 8, **PCOS**—Polycystic Ovary Syndrome, **PDXOs**—Patient-Derived Xenograft Organoids, **PIFO**—Primary Ciliary Dyskinesia Protein FOXJ1-Associated, **PR**—Progesterone Receptor, **PR-B**—Progesterone Receptor B, **PRDX4**—Peroxiredoxin 4, **PRKAR1A**—Protein Kinase cAMP-Dependent Type I Regulatory Subunit Alpha, **PROM1**—Prominin 1 (CD133), **qPCR**—Quantitative Polymerase Chain Reaction, **ROCK inhibitor**—Rho-associated Kinase inhibitor, **RPL**—Recurrent Pregnancy Loss, **RSPH1**—Radial Spoke Head 1, **RSPO1**—R-spondin 1, **SNORD14E**—Small Nucleolar RNA, C/D Box 14E, **SOX9**—SRY-Box Transcription Factor 9, **SOX17**—SRY-Box Transcription Factor 17, **SP**—Seminal Plasma, **SSEA**-1—Stage-Specific Embryonic Antigen-1, **TeloCol**-6—Telopeptide-Depleted Collagen Type I, **TepP**—Type III secreted effector protein, **TGF-β**—Transforming Growth Factor Beta, **UEM**—Uterus Extracellular Matrix, **UO**—Urothelial Organoids, **VEGFA**—Vascular Endothelial Growth Factor A, **WDR16**—WD Repeat Domain 16.
